# Functional Redundancy of DICER Cofactors TARBP2 and PRKRA During Murine Embryogenesis Does Not Involve miRNA Biogenesis

**DOI:** 10.1534/genetics.118.300791

**Published:** 2018-02-21

**Authors:** Sri Ramulu N. Pullagura, Bill Buaas, Nichelle Gray, Lindsey C. Krening, Anuj Srivastava, Robert E. Braun

**Affiliations:** * The Jackson Laboratory, Bar Harbor, Maine 04609; † Graduate School of Biomedical Sciences and Engineering, The University of Maine, Orono, Maine 04469

**Keywords:** TARBP2, PRKRA, PACT, DICER, miRNA

## Abstract

Several *in vitro* studies have suggested that canonical microRNA (miRNA) biogenesis requires the DICER cofactors TARBP2 and PRKRA for processing of pre-miRNAs to mature miRNAs. To investigate the roles of TARBP2 and PRKRA in miRNA biogenesis *in vivo*, and to determine possible functional redundancy, we first compared the phenotypes of *Tarbp2* and *Prkra* single and double mutants. In contrast to *Dicer ^−/−^* embryos, which die by embryonic day 7.5 (E7.5), single *Tarbp2 ^−/−^* and *Prkra ^−/−^* mice survive beyond E7.5 and either die perinatally or survive and exhibit cranial/facial abnormalities, respectively. In contrast, only a few *Tarbp2 ^−/−^*; *Prkra ^−/−^* double mutants survived beyond E12.5, suggesting genetic redundancy between *Tarbp2* and *Prkra* during embryonic development. Sequencing of miRNAs from single-mutant embryos at E15.5 revealed changes in abundance and isomiR type in *Tarbp2 ^−/−^*, but not *Prkra ^−/−^*, embryos, demonstrating that TARBP2, but not PRKRA, functions in miRNA biogenesis of a subclass of miRNAs, and suggesting that functional redundancy between TARBP2 and PRKRA does not involve miRNA biogenesis.

MICRORNAS (miRNAs) are a large family of endogenously encoded ∼22 nucleotide (nt) noncoding RNAs that modulate post-transcriptional gene regulation through translational control and the decay of messenger RNAs (mRNAs) (Krol *et al.* 2010). Target specificity of a miRNA is conferred by the seed sequence, which comprises nucleotides 2–8 of the ∼22-nt small RNA (Lewis *et al.* 2003, 2005; Doench and Sharp 2004; Grimson *et al.* 2007; Wee *et al.* 2012). Extensive base pairing of the miRNA seed sequence to the target mRNA leads to translational repression and mRNA degradation, with ∼50% of all protein-coding genes predicted to be miRNA targets (Baek *et al.* 2008; Selbach *et al.* 2008; Friedman *et al.* 2009). The human genome is estimated to encode ∼1900 miRNA genes, with many exhibiting developmental and stage-specific expression patterns (Landgraf *et al.* 2007; Griffiths-Jones *et al.* 2008).

Mammalian DICER is the principal cytoplasmic enzyme that generates miRNAs and, in association with its double-stranded RNA-Binding Protein (dsRBP) cofactors TARBP2 and PRKRA, binds the ∼70-nt-long pre-miRNA “hairpin” and cleaves the loop to produce a double-stranded RNA (dsRNA) duplex (Bernstein *et al.* 2001; Grishok *et al.* 2001; Hutvagner *et al.* 2001; Ketting *et al.* 2001; Knight and Bass 2001; Chendrimada *et al.* 2005; Haase *et al.* 2005; Lee *et al.* 2006; Kok *et al.* 2007; Koscianska *et al.* 2011). The fully processed duplex RNA is loaded into the RNA-induced silencing complex (RISC), where one strand is degraded leaving the guide strand (Kawamata and Tomari 2010). In TARBP2, the double-stranded RNA-binding domain (dsRBD) motif is present in two copies along with a C-terminal protein–protein “Medipal” interaction domain that binds DICER (Lee *et al.* 2004; Laraki *et al.* 2008; Daniels *et al.* 2009). Several *in vitro* studies have shown that the DICER-TARBP2 complex exhibits ATP-independent dynamic diffusion along the length of pre-miRNA until it encounters a hairpin structure, which determines the site of cleavage by DICER (Koh *et al.* 2012, 2017; Wilson *et al.* 2015). *Prkra* is the only mammalian *Tarbp2* paralog and encodes a protein with high sequence similarity and structure to TARBP2. Like TARBP2, PRKRA contains two dsRBDs and a C-terminal Medipal domain that interacts with DICER (Laraki *et al.* 2008; Daniels and Gatignol 2012). *In vitro* biochemical and cell culture experiments suggest that loss of the DICER cofactors TARBP2 and PRKRA disrupts pre-miRNA processing, RISC loading of miRNAs, and RNA interference (RNAi) activity (Chendrimada *et al.* 2005; Haase *et al.* 2005; Chakravarthy *et al.* 2010; Daniels and Gatignol 2012). Apart from miRNA biogenesis, TARBP2 is reported to bind dsRNA directly and destabilize it (Goodarzi *et al.* 2014), whereas PRKRA regulates translation by activating protein kinase (PKR) (Daniels and Gatignol 2012), a global inhibitor of translation. TARBP2 also promotes translation by binding and inhibiting PKR during human immunodeficiency virus infection, thereby assisting in viral replication (Daniels and Gatignol 2012).

Maternally supplied *Dicer* mRNA is expressed in murine oocytes, reaching its highest levels in germinal vesicle-stage oocytes, and then falling to its lowest detectable level in eight-cell-stage embryos (Murchison *et al.* 2007). Conditional ablation of *Dicer* in oocytes using *Zp3Cre* results arrest in meiosis I as a consequence of spindle dysfunction and defects in chromosome congression (Murchison *et al.* 2007; Tang *et al.* 2007). Loss of zygotic *Dicer* mRNA, which normally begins to be expressed in blastocyst-stage embryos (Murchison *et al.* 2007), leads to early developmental defects and embryonic arrest shortly after gastrulation around embryonic day 7.5 (E7.5) (Bernstein *et al.* 2003). Furthermore, conditional gene targeting has revealed that DICER functions in the development or homeostasis of embryonic and fetal organs including the cardiovascular, genitourinary, musculoskeletal, and nervous systems (Bernstein *et al.* 2003; O’Rourke *et al.* 2007; Saal and Harvey 2009; Zehir *et al.* 2010; Small and Olson 2011). These data are consistent with a model where miRNAs function in the proper development of numerous mammalian organs, and disruptions in this small RNA biogenesis pathway can result in congenital birth defects and in extreme cases fetal death. The mouse *Tarbp2* gene encodes a 365-amino acid protein that is localized predominantly to the cytoplasm, and *Tarbp2 ^−/−^* mice on a hybrid background are viable but have reduced body size and are male sterile (Zhong *et al.* 1999). Previously characterized *Prkra* mutant mice are homozygous viable with cranial/facial defects (Rowe *et al.* 2006; Dickerman *et al.* 2011) and, like *Tarbp2* mutants, exhibit postnatal growth retardation on several genetic backgrounds, including C57BL/6J. The similar but different phenotypes of *Tarbp2 ^−/−^* and *Prkra ^−/−^* mutants led us to investigate their role as DICER cofactors *in vivo*.

## Materials and Methods

### Mouse mutants

*Tarbp2^tm1reb^* (Zhong *et al.* 1999) mice were genotyped as described previously. Genotyping for the *Prkra ^lear1J^* mice was carried out as recommend by the Jackson Laboratory mutant repository.

### High-throughput sequencing of fetal small RNAs

E15.5 C57BL/6J-*Tarbp2 ^tm1reb^* and C57BL/6J-*Prkra ^lear1J^* embryos were generated through timed mating intercrosses of heterozygous male and female mutant mice. Embryos were dissected at 15.5 days postcoitum and the associated yolk sac was used for genotyping. The developmental stage was confirmed using Theiler staging criteria for mouse embryo development. Embryos were decapitated and both body and head were immediately immersed in RNAlater (Thermo Fisher). Whole fetuses (head and body) were homogenized in TRIzol followed by purification of total RNA using a QIAGEN miRNeasy kit (Valencia, CA). High-throughput sequencing libraries containing small noncoding RNAs (*e.g.*, miRNAs or Piwi-interacting RNAs) were generated using 1 μg of total RNA and an Illumina TruSeq small RNA library preparation kit (Illumina, Inc.). Individually barcoded fetal libraries were pooled and sequenced (100 bp-end reads) on a single HiSequation 2000 flow cell lane running version 3 chemistry. Illumina CASAVA software was used to carry out BCL to Fastq conversion. All the samples were passed through quality control and sequences with poor base qualities were removed. The adaptor sequences were removed using the clip_adaptors.pl module of mirdeep (v2.0.0.5) (Friedlander *et al.* 2012). Raw reads were collapsed based on sequence identity using the collapse_reads_md.pl module of mirdeep2. Collapsed reads were further annotated for miRNA by mapping to miRBase version 21 (Griffiths-Jones *et al.* 2008; Kozomara and Griffiths-Jones 2011; Friedlander *et al.* 2012) using the miraligner module of seqbuster (Pantano *et al.* 2010). IsomiRs were identified using the R isomiRs (v1.3.0) package of seqbuster. Pairwise differential expression was performed among different groups using DESEQ2 (v1.12.4) (Love *et al.* 2014).

### Quantitative RT-PCR and Taqman assays

Adult tissues were used for total RNA extraction (TRIzol; Invitrogen, Carlsbad, CA), followed by the production of random primed cDNA (18080051; Invitrogen). Real-time PCR using SYBR green (ABI 7500; Thermo Fisher) and the ddCT method for calculating relative gene expression were used to determine *Prkra* and *Tarbp2* transcript levels. *b-actin* amplification was used as an endogenous control. For miRNA Taqman assays, total RNA is extracted (TRIzol extraction; Invitrogen) from transformed mouse embryonic fibroblasts (MEFs), followed by production of cDNA specific to each miRNA (4427975; Thermo Fisher) using a Taqman miRNA reverse transcription kit (4366596; Thermo Fischer). Taqman PCR using Taqman Universal Master Mix II (4440042; Thermo Fisher) and the ddCT method for calculating relative transcript expression were used to determine mature miRNA levels. U6 amplification was used as an endogenous control.

### Western blotting

PRKRA and TARBP2 protein levels were assayed from the tissues and MEFs using standard western blotting protocols. A rabbit monoclonal antibody raised against a synthetic peptide corresponding to the C-terminal end of human PRKRA (#ab75749; Abcam) and a TARBP2 antibody from a previous study (Zhong *et al.* 1999) were used to quantify protein levels. GAPDH was used as a loading control.

### Cell culture, transformation, and cell number calculation

MEFs were isolated at E11.5 and transformed by infecting with viral supernatant from simian virus 40 (SV40) T antigen (pBABE SV40 T antigen from Addgene #13970)-packaged cells (Plat-E ecotropic packaging). To obtain stably transfected clones, cells were selected with 2 μg/ml puromycin and single clones were isolated using clonal rings. Transformed MEFs were cultured in Dulbecco’s modified Eagle’s medium (DMEM containing high glucose level; GIBCO [Grand Island Biological], Grand Island, NY) supplemented with 10% fetal bovine serum (Invitrogen). For cell number quantification, transformed MEFs were seeded at an initial density of 1 × 10^5^ cells/well on the day prior to treatment with 40 mg/ml DMSO, enoxacin, and incubated at 37° in 5% CO_2_. The cell numbers were calculated on day 0, day 3, and day 5 using trypan blue staining and a Countess cell counter.

### Embryo dissection and β-gal staining

Timed matings were set up to collect the embryos varying from embryonic day E8.5 to E13.5. Embryo genotypes were determined by PCR from yolk sac DNA. Embryos were fixed in 4% paraformaldehyde and stained overnight in 5-bromo-4-chloro-3-indolyl--D-galactoside-containing solution to visualize β-galactosidase activity derived from the lacZ gene tag in the *Tarbp2*-targeted mutation. For tissue sections, embryos were frozen in optimum cutting temperature (OCT) compound (Sakura Finetek USA Inc.) and cryostat sectioned. Sections were fixed (0.2% gluteraldehyde) and stained as above. Sections were postfixed, counterstained with Nuclear Fast Red, mounted, and imaged under an Olympus Nanozoomer with the desired magnification.

### Skeletal staining and histology

For ossified bone and cartilage staining, E15.5 embryos were processed using an alizarin red/alcian blue standard staining protocol (Hogan *et al.* 1994). Embryos collected at E18.5 were fixed for histology in Bouin’s fixative, then paraffin embedded. Next, 10 μm serial sections were processed using hematoxylin and eosin staining.

### Data availability

Strains are available upon request. miRNA sequencing data has been uploaded to Bioproject ID: PRJNA423238: *Mus musculus*. Raw sequence reads are available at the Sequence Read Archive (SRA): (TaxId: 39442) SRA ID: SRP127346.

## Results

### *Tarbp2* is broadly expressed during embryonic development

To determine where *Tarbp2* is expressed during embryonic development, we analyzed β-galactosidase expression in heterozygous *Tarbp2 ^tm1reb/+^* embryos (herein referred to as *Tarb2 ^−/+^*) containing a LacZ insertion that transcriptionally tags the *Tarbp2* gene (Supplemental Material, Figure S1A). In whole-mount staining of *Tarbp2 ^+/−^* embryos, we observed high levels of β-galactosidase activity throughout all stages of development, as early as E8.5 when *Dicer* is expressed (Figure S1B). In sections of E13.5 organs, we detected expression in some, but not all, cells in the heart, hindbrain, and liver (Figure S1C). This organ expression was confirmed by RT-PCR analysis of *Tarbp2* in wild-type E13.5 extracts (Figure S1C). *Tarbp2* is expressed in all adult mouse tissues, with abundant transcript levels in the testis, although expression is restricted by cell type (Zhong *et al.* 1999). Thus, mouse *Tarbp2* is broadly expressed from early embryonic stages through to adulthood with likely cell type-specific expression in many, if not all, tissues.

### *Tarbp2* null animals are smaller but exhibit no gross anatomical defects

Previously, we reported a smaller body size, early postnatal lethality, and male sterility in *Tarbp2* ^−/−^ mice on a mixed genetic background (Zhong *et al.* 1999). To determine if early defects in ossification could explain the size difference in mutant animals, we stained E15.5 embryos with alizaran red/alcian blue to mark bone and cartilage, respectively. Stained embryos were smaller than control littermates (Figure S1D) with delayed ossification of the tympanic membrane and spinal column. To compare the size of organs and soft tissues in *Tarbp2* mutants, we performed micro computer tomography (microCT) on E15.5 embryos. We observed a 20% decrease in total volume in *Tarbp2* mutants compared to controls (red outline, Figure S1E) but no significant relative difference in the size of any single organ or tissue. We conclude that growth retardation defects begin *in utero*, as early as E15.5.

### The *Tarbp2* null allele is embryonic lethal on the C57BL/6J mouse strain

*Tarbp2 ^−/−^* mice have reduced body mass and perinatal lethality on a mixed strain background (Zhong *et al.* 1999). Because the genetic background can impact phenotypic variation, we tested whether strain background had an effect on the phenotypes observed in *Tarbp2* mutants, and therefore compared the frequency of observed homozygotes on the 129S4 (129) background to the C57BL/6J (B6) background. In heterozygous (het) × het breeding crosses in the 129 background, we observed a significant reduction in the number of *Tarbp2 ^−/−^* homozygotes at P21, suggesting reduced viability on the 129 background (Table 1). *Tarbp2 ^−/−^* mutants on the B6 genetic background exhibited an even more extreme phenotype. In het × het crosses, we were unable to recover any homozygous animals at P14. Furthermore, at E17.5, we observed only 13% of the expected 25% homozygous animals. In crosses between 129 *Tarbp2 ^+/−^* females and B6 *Tarbp2 ^+/−^* males, we observed the expected Mendelian ratios at P21, suggesting that the 129 genetic background has one or more modifiers that can suppress the lethality observed on the B6 background.

**Table 1:** Background strain dependence of *Tarbp2*

Strain	Age	+/+	+/−	−/−
129S4 (129)	P21	29 (26%)	66 (58%)	18 (16%)
C57Bl/6J (B6)	E17.5	11 (21%)	35 (66%)	7 (13%)
	P14	23 (31%)	53 (69%)	0 (0%)
129 × B6 F1	P21	22 (23%)	51 (53%)	23 (24%)

P, postnatal day; E, embryonic day.

### *Prkra^1J^* mutants are viable with anatomical defects

Like TARBP*2*, PRKRA binds DICER and has been shown to influence miRNA biogenesis *in vitro*. To compare the requirements for *Tarbp2* and *Prkra* during development, we next analyzed *Prkra* mutant mice. Three spontaneous mutant *Prkra* alleles (*lear*, *little ear*) have arisen in The Jackson Laboratory repository colony, displaying small body size and reduced ear size. These phenotypes are similar to the previously characterized *Prkra*-targeted mutant (Rowe *et al.* 2006) and chemically induced mutants (Dickerman *et al.* 2011) that produce a complete PRKRA protein deficiency or a protein isoform deficient in dsRNA binding, respectively. The *lear1J* allele (referred to herein as *Prkra ^−^*) contains a splice donor mutation in intron 5 (Figure S2A). Using RT-PCR on RNA from E13.5 *Prkra ^−/−^* mutant organ extracts, we found a significant decrease in *Prkra* expression in heart and kidney (Figure S2B). Furthermore, immunoblots for PRKRA protein showed dramatically decreased levels in liver, lung, kidney, and spleen, with complete loss of protein in the heart (Figure S2C). Thus, *Prkra^1J^* is a strong hypomorphic or null allele that phenocopies previously characterized spontaneous and targeted alleles.

### *Tarbp2 ^−/−^*; *Prkra ^−/−^* double mutants die by midgestation

**Figure 1:**
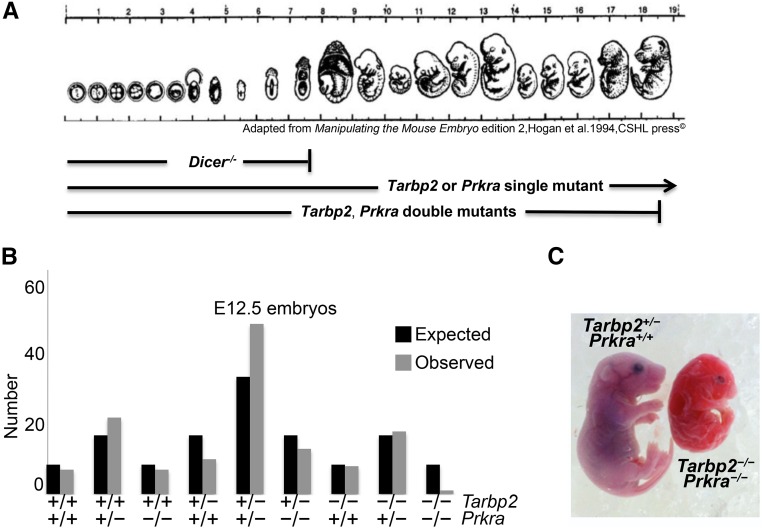
*Tarbp2* ^−/−^;*Prkra ^−/−^* mutants die at midgestation. (A) An image summarizing the developmental stages of survival or embryonic lethality of *Dicer ^−/−^*, *Tarbp2* ^−/−^, or *Prkra* ^−/−^ single mutants and *Tarbp2* ^−/−^; *Prkra* ^−/−^ double mutants. (B) Number of embryonic day (E)12.5 embryos harvested from crossing *Tarbp2 ^−/+^*; *Prkra ^−/+^* double heterozygotes. χ^2^ = 19.283 (8 d.f.), two-tailed *P*-value = 0.0134. (C) An E18.5 *Tarbp2* ^−/−^;*Prkra ^−/−^* mutant that is significantly smaller than a *Tarbp2* ^+/−^;*Prkra ^+/+^* sibling, and has a shorter snout.

Because *Dicer* mutants die by E7.5 (Figure 1A), while *Tarbp2 ^−/−^* mutants die perinatally, and *Prkra* mutants are viable but have reduced body size and ear deformities, we asked whether there might be functional redundancy between *Tarbp2* and *Prkra* during early embryogenesis. To test this, we crossed *Tarbp2 ^+/−^*; *Prkra ^+/−^* double heterozygotes and genotyped animals at birth. We failed to obtain any *Tarbp2 ^−/−^*; *Prkr ^−/−^* (B6) animals. We therefore looked at earlier embryonic time points and recovered one E18.5 double mutant that was half-the-size of control littermates and exhibited severe shortening of the snout (Figure 1C). Additionally, the heart was smaller and there were open cranial sutures (data not shown). This animal was able to survive quite late considering that we were only able to recover one double mutant out of an expected eight, at E12.5 (Figure 1B, chi square *P* < 0.05). To determine if open cranial features were a hallmark of *Prkra* mutants, and if they are affected by the *Tarbp2* locus, we analyzed cranial morphology using microCT in 8-week-old animals. We consistently observed open cranial sutures in *Prkra ^−/−^* mutants, and reduction in *Tarbp2* gene dosage further exacerbated this phenotype, as exemplified in *Tarbp2 ^−/+^*; *Prkra ^−/−^* mutants (Figure S2D). These data indicate that the structurally similar dsRBPs, TARBP2 and PRKRA, genetically interact.

### miRNA processing is altered in *Tarbp ^−/−^* but not *Prkra ^−/−^* mutants

**Figure 2:**
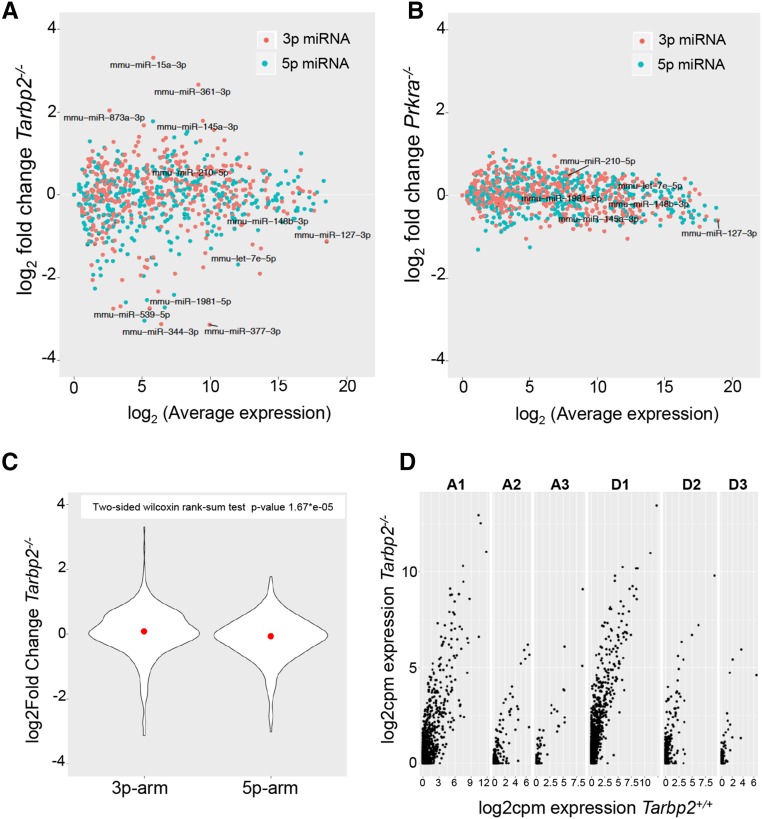
*Tarbp2* regulates processing of a subclass of pre-miRNAs (microRNAs). (A) Plot of miRNA transcripts in *Tarbp2 ^−/−^* whole embryos at embryonic day (E)15.5 and their fold-changes by average expression using RNA-sequencing (RNA-seq). (B) Plot of miRNA transcripts in *Prkra ^−/−^* whole embryos at E15.5 and their fold-changes by average expression using RNA-seq. (C) Plot of overall expression fold-change of 3p and 5p miRNA transcripts in C57Bl6 *Tarbp2 ^−/−^* whole embryos at E15.5 compared to wild-type embryos. (D) Plot of 3p arm miRNA transcripts with one, two, or three nucleotide (*n* = 1, 2, or 3) addition (An) or deletion (Dn) at the 5′ end in *Tarbp2 ^−/−^* whole embryos at E15.5 and their fold-changes by counts per million (cpm) using RNA-seq.

The difference in phenotypes between *Dicer*, *Tarbp2*, and *Prkra* single and double mutants led us to ask whether TARBP2 and PRKRA influenced DICER-mediated miRNA biogenesis *in vivo*. We collected total RNA from E15.5 B6 *Tarbp2 ^−/−^* and *Prkra* ^−/−^ embryos and generated high-throughput sequencing libraries of small noncoding RNAs for RNA-sequencing (RNA-seq). Using a false discovery rate pass of < 0.05, we found that the expression levels of 74 mature miRNAs were significantly changed in *Tarbp2 ^−/−^* mutants (Figure 2A). Interestingly, these transcripts were both decreased (*n* = 46, 62%) and increased (*n* = 28, 38%), suggesting that TARBP2 is required for a subpopulation of miRNAs during this stage of development. Conversely, there was no change in the expression of miRNAs in *Prkra ^−/−^* embryos (Figure 2B), suggesting that PRKRA has no discernable role in miRNA biogenesis during this stage of development.

**Figure 3:**
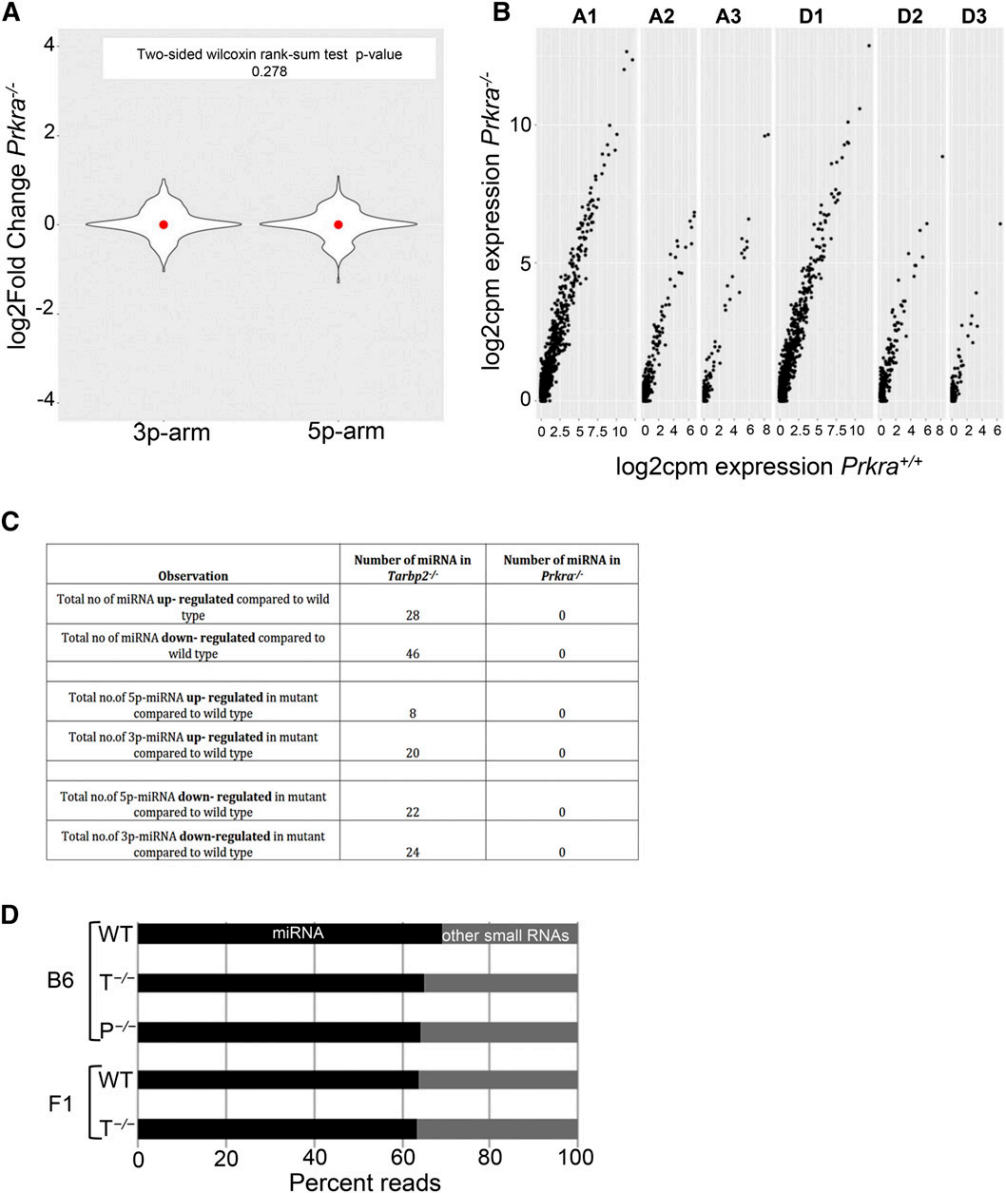
*Prkra* is not required for canonical microRNA (miRNA) biogenesis at embryonic day (E)15.5: (A) Fold-change of overall expression of 3p and 5p miRNA transcripts in C57Bl6 *Prkra ^−/−^* whole embryos at E15.5 compared to wild-type embryos. (B) Plot of 3p arm miRNA transcripts with one, two, or three nucleotide (*n* = 1, 2, or 3) addition (An) or deletion (Dn) at the 5′ end in *Prkra ^−/−^* whole embryos at E15.5 and their fold-changes by counts per million (cpm) using RNA-sequencing. (C) A table chart summarizing the type of deregulation and number of miRNAs in *Tarbp2 ^−/−^* and *Prkra ^−/−^* compared to wild-type. (D) Chart of percent reads of miRNAs *vs.* other small noncoding RNAs in the RNA-sequencing libraries per genotype and background. P*^−/−^*, *Prkra ^−/−^*; T*^−/−^*, *Tarbp2 ^−/−^*; WT, wild-type.

Biochemical data suggests that the dsRBPs can influence strand selection of the processed pre-microRNA. As TARBP2 determines the site of cleavage by DICER, and addition or deletion of a nucleotide can alter the stability of processed miRNA arms, we compared the overall expression level changes of 3p-miRNAs and 5p-miRNAs. Significant expression level changes were observed in both populations; however, there were more changes to 3p-miRNAs (44 out of 74) than 5p-miRNAs (30 out of 74) in *Tarbp2 ^−/−^* embryos compared to wild-type embryos, suggesting a preferential defect in processing of 3p-miRNAs in the absence of TARBP2 (Figure 2C). Also, in *Tarbp2 ^−/−^* embryos we observed that there was an increase in the number of reads mapped to 3p-miRNAs with an addition (A_n_) or deletion (D_n_) of nucleotides at the 5′ end compared to wild-type embryos (Figure 2D), suggesting an improper cleavage site selection by DICER in the absence of TARBP2. In *Prkra ^−/−^* mutant embryos, the level of expression of 3p-miRNAs and 5p-miRNAs remained the same as in wild-type embryos (Figure 3A), as did the length of the 3p-miRNAs (Figure 3B). A comparison of the observed changes in miRNA populations in *Tarbp2 ^−/−^* and *Prkra ^−/−^* mutants is shown in Figure 3C. Because an equal number of reads were mapped to miRNAs and other small noncoding RNAs independent of the genotype (Figure 3D), we conclude that TARBP2 but not PRKRA is required for the processing of a subclass of miRNAs at E15.5.

### Pharmacological assessment of the role of TARBP2 and PRKRA on miRNA processing

**Figure 4:**
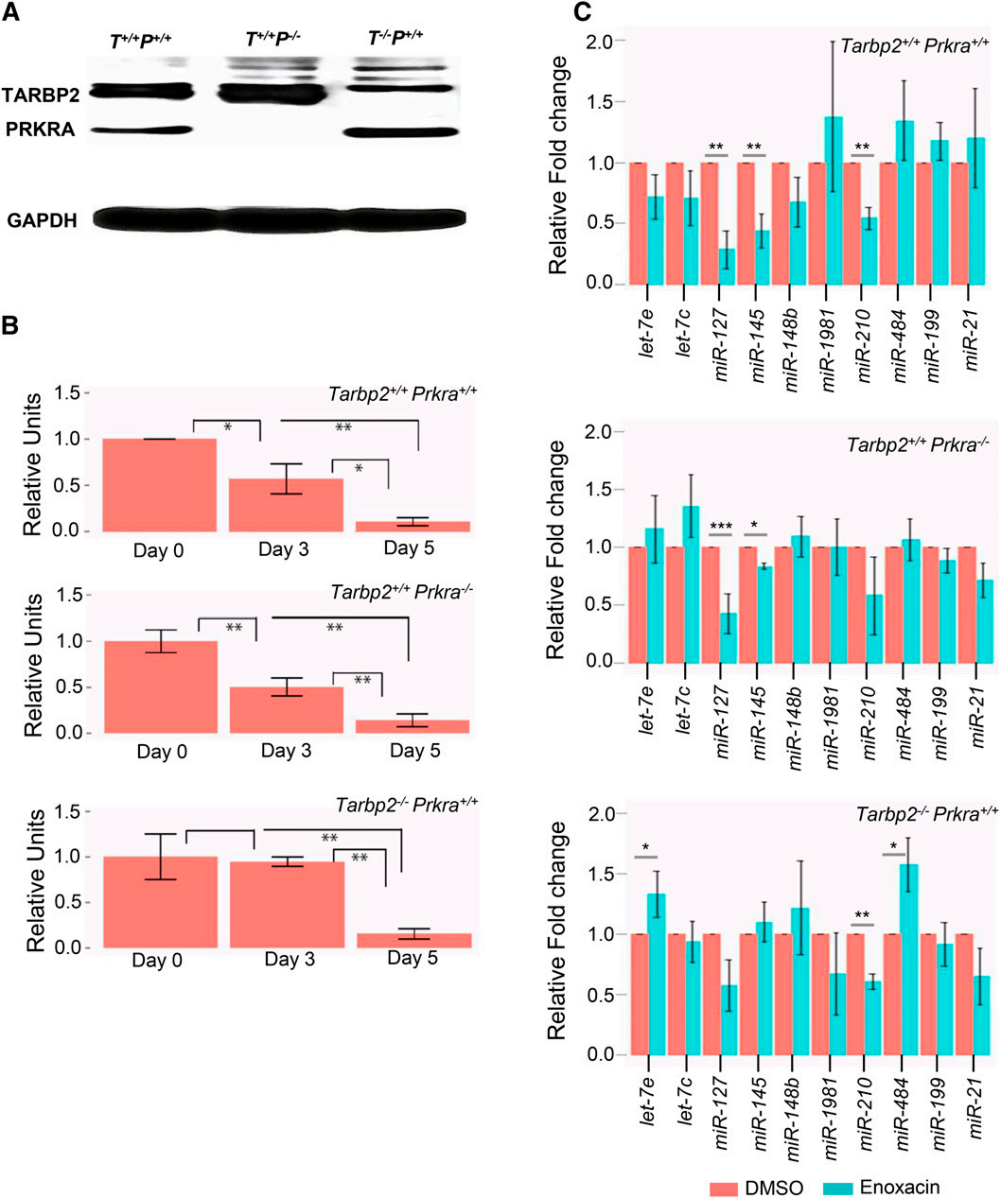
Enoxacin has its effect on transformed mouse embryonic fibroblasts (MEFs) independent of *Tarbp2* or *Prkra*: (A) Immunoblot for TARBP2 and PRKRA shows the expression or absence of these proteins based on their genotype in transformed MEFs. (B) Graphical representation of cell numbers for the indicated genotype after enoxacin treatment from day 0 to day 5. Relative units are obtained after normalizing the cell numbers from enoxacin treatment to the cell numbers with DMSO treatment (* *P* < 0.05 and ** *P* < 0.005). (B) Expression fold-change of 10 quantified mature microRNAs (miRNAs) in transformed MEF cell lines with the indicated genotype upon enoxacin treatment (* *P* < 0.05 and ** *P* < 0.005).

In a screen for small molecules that modify RNAi activity, the synthetic antibacterial compound enoxacin was identified as an enhancer of RNAi (Shan *et al.* 2008). Several studies have reported that enoxacin binds human TARBP2, increases TARBP2 affinity for pre-miRNAs, and enhances the processing of pre-miRNAs to mature miRNAs (Melo *et al.* 2011; Cornaz-Buros *et al.* 2014). To confirm our findings that TARBP2, but not PRKRA, is involved in miRNA processing during embryogenesis, we examined the effect of enoxacin on the growth of MEFs derived from *Tarpb2 ^−/−^* and *Prkra ^−/−^* mutant embryos. Western blot analysis showed that PRKRA and TARBP2 were detected in MEFs derived from wild-type embryos but not *Prkra ^−/−^* or *Tarbp2 ^−/−^* mutants, respectively (Figure 4A). To assess whether enoxacin differentially affected *Prkra ^−/−^* but not *Tarbp2 ^−/−^* mutant MEFs, we treated the cells with DMSO and enoxacin as described previously (Melo *et al.* 2011). When cell numbers from day 0, day 3, and day 5 were plotted comparing DMSO-treated cells with enoxacin, we observed that *Tarbp2 ^−/−^* mutants were more resistant to enoxacin than wild-type and *Prkra ^−/−^* mutant MEFs at day 3, although by day 5 all genotypes were similarly affected (Figure 4B and Figure S3). To directly test the effect of enoxacin on miRNA levels, we measured the effect of enoxacin on the abundance of 10 miRNAs whose levels were altered in *Tarbp2 ^−/−^* E15.5 embryos. Treatment of wild-type MEFs with enoxacin significantly decreased the relative levels of 3/8 miRNAs (Figure 4C top), although none of the levels of miRNAs were elevated as we had predicted. The levels of two of the miRNAs, miR-127 and miR145, remained significantly depressed in *Prkra ^−/−^* MEFs, while the levels of one miRNA, miR-120, were rescued, although not to wild-type levels (Figure 4B middle). On the other hand, mutation of *Tarbp2* rescued levels of miR-127 and miR145 to wild-type levels, but had no effect on miR-120 and actually resulted in an increase in two miRNAs, let-7e and miR-484, that had been unaffected in wild-type MEFs. From these observations we conclude that enoxacin does act solely through TARBP2 and that there is no direct correlation with the increase in miRNAs and the effect of enoxacin on the cell growth of transformed MEFs.

## Discussion

Previous *in vitro* studies suggested that TARBP2 and PRKRA act as cofactors of DICER for the processing of pre-miRNA to mature-miRNAs. The discordance between the phenotypes of *Dicer1 ^−/−^*, *Tarbp2 ^−/−^*, and *Prkra ^−/−^* single mutants led us to test these findings and to determine if TARBP2 and PRKRA have redundant functions in miRNA biogenesis.

Our results strongly suggest that *Tarbp2* and *Prkra* genetically interact during embryonic development. Earlier studies have established the essentiality of *Dicer* during oogenesis and embryogenesis. Maternal Dicer mRNA, which is present throughout oocyte development (Murchison *et al.* 2007), is required for the completion of meiosis 1 (Murchison *et al.* 2007; Tang *et al.* 2007), and elimination of zygotic *Dicer* expression results in an embryonic lethality phenotype by E7.5. We failed to recover the expected number of *Tarbp2 ^−/−^*, *Prkra ^−/−^* double mutants at E12.5, and recovered only one double-mutant embryo, which was severely abnormal, at E18.5. It is possible that the missing embryos died prior to E7.5, similar to that seen for *Dicer^−/−^* embryos, and that the few embryos recovered at E12.5 and E18.5 could be due to incomplete penetrance of the double-mutant phenotype. Furthermore, we found that the allele status at *Tarbp2* affected the closure of cranial sutures observed in *Prkra ^−/−^* mutants.

miRNA sequencing of wild-type and single mutants at E15.5 revealed that TARBP2, but not PRKRA, is essential for proper miRNA biogenesis of a set of miRNAs during embryonic development. We chose to analyze E15.5 embryos because of the lack of an observable phenotype in single mutants at that time point and because double-mutant embryos were absent, suggesting a requirement for both TARBP2 and PRKRA at that stage. We observed changes in the abundance and length of both 3p and 5p miRNAs in *Tarbp2 ^−/−^* mutants, although the extent of aberrant processing of 3p-miRNAs was greater than for 5p-miRNAs. Our findings for TARBP2 are supported by a recently published study using knockout cell lines (Kim *et al.* 2014). We did not observe differences in the abundance, sequence, or lengths of miRNAs in *Prkra ^−/−^* mutants, suggesting that PRKRA is not involved in miRNA biogenesis during murine embryogenesis. However, given that we were able to detect minor levels of PRKRA in some tissues (Figure S2), although not in MEFs (Figure 4), it remains an open possibility that PRKRA is involved in miRNA biogenesis.

Interestingly, the absence of TARBP2 affected some but not all miRNAs. Of the 74 miRNAs whose levels were affected, 28 were increased and 46 were decreased. We do not know the basis for the selectivity. In addition, because we used RNA isolated from whole embryos, we do not know if the affected miRNAs are expressed in the same cells or in different cells. These observations support the hypothesis that DICER acts independently of TARBP2 prior to E7.5, as not all miRNAs require TARBP2 for their biogenesis. We also observed a dependency for TARBP2 on isomiR biogenesis with a bias toward a stronger effect on 3p-miRNAs, as previously reported (Wilson *et al.* 2015). The effect of this change could alter the half-life or stability of improperly processed mature miRNAs and ultimately result in the deregulation of RISC complex formation. Improper targeting of mRNAs by isomiRs could result in the developmental defects observed in *Tarbp2 ^−/−^* animals. The viability of B6129F1 *Tarbp2 ^−/−^* hybrids may also be explained by the restricted effect of the loss of TARBP2 on miRNA biogenesis. Heterozygosity at miRNA and target mRNA loci across the genome may alter the global miRNA/mRNA profile and suppress the relatively minor changes in miRNA levels and isomiR types observed in *Tarbp2 ^−/−^* mutants. In support of this, we attempted to identify quantitative trait loci in the 129 strain that suppress the lethality and growth defects observed in B6. Assaying for both viability and body weight, we mapped several loci with modest LOD scores across the genome that correlated with one or both phenotypes, indicating that there are multiple loci that contribute to enhanced survival and body weight in the F1 hybrid, N2, or mixed backgrounds (Figure S4).

In an attempt to pharmacologically strengthen our findings, we assessed the effect of enoxacin on MEFs derived from *Tarbp2 ^−/−^* and *Prkra ^−/−^* mutant embryos. Enoxacin has been previously reported to inhibit the growth of cancer cells by enhancing miRNA processing through a direct physical interaction with TARBP2 miRNA (Shan *et al.* 2008; Melo *et al.* 2011; Cornaz-Buros *et al.* 2014). If TARBP2 and PRKRA are redundant, then cells expressing either of these proteins should have a similar response to enoxacin treatment. If enoxacin acts only through TARBP2, then *Tarbp2 ^−/−^*, but not *Prkra ^−/−^*, mutant MEFs should be resistant to its effects. We initially found that the growth of transformed *Tarbp2 ^−/−^* MEFs was more sensitive to enoxacin than *Prkra ^−/−^* MEFs, supporting the possibility that enoxacin may act through TARBP2. However, molecular analysis failed to reveal an increase in miRNA levels in any genotype, and the changes that were observed were the opposite of what has been observed in cancer cells. Based on these observations, we conclude that the growth effects of enoxacin on transformed MEFs are independent of TARBP2 or PRKRA and that enoxacin may act through a mechanism in transformed MEFs that is different to that in cancer cells.

The discordant phenotypes between *Dicer* and *Tarbp2* mutants, and the observation that only a subset of miRNAs are affected in *Tarbp2* mutants, suggest that DICER does not require a cofactor for processing some pre-miRNAs or that another as yet unidentified cofactor of DICER functions in early embryogenesis. It has recently been shown that DICER interacts with ADAR and lack of this association in *Adar1* mutants impacts the biogenesis of mature miRNAs at E11.5 (Ota *et al.* 2013). These combined results suggest that DICER interacts with different dsRBPs in canonical miRNA biogenesis during embryonic development and that this association is specific to developmental stage. DICER may also perform a function in early embryogenesis that is independent of miRNA biogenesis, as has been previously suggested (Johanson *et al.* 2013).

The failure to detect defects in miRNA biogenesis in *Prkra* mutants at E15.5, and the discordant phenotypes between *Dicer* single mutants and *Tarbp2 ^−/−^*; *Prkra ^−/−^* double mutants, suggests that the double-mutant phenotype is not solely due to defects in miRNA biogenesis. The severity of the phenotype in *Tarbp2 ^−/−^*; *Prkra ^−/−^* double-mutant embryos could be due to the combination of defects in miRNA biogenesis, as a consequence of the absence of TARBP2, coupled with defects in endo-siRNA (small interfering RNA) biogenesis as a consequence of the absence of PRKRA. That said, deletion of *Dicer* failed to affect the ability of siRNAs to repress gene expression in murine embryonic stem cells (Murchison *et al.* 2005). Alternatively, TARBP2 and PRKRA could be functioning in a shared pathway that is independent of DICER-mediated pre-miRNA processing. TARBP2 has been shown to act independently of DICER to destabilize dsRNA (Goodarzi *et al.* 2014), while PRKRA regulates translation by activating PKR (Daniels and Gatignol 2012), a global regulator of translation. The absence of both proteins could result in increased levels of dsRNA, hyper-activation of PKR, and loss of global control of translation leading to broad inhibition of development.

## Supplementary Material

Supplemental material is available online at www.genetics.org/lookup/suppl/doi:10.1534/genetics.118.300791/-/DC1.
