# Supplementary material for: Functional Redundancy of DICER Cofactors TARBP2 and PRKRA During Murine Embryogenesis Does Not Involve miRNA Biogenesis
Source: Genetics. 2018 Feb 21;208(4):1513–22. doi: 10.1534/genetics.118.300791 (PMC5887145; doi:10.1534/genetics.118.300791)

Supplemental_Figure_S1, related to Fig. 1. (A) Schematic representation of the *Tarbp2* ^{tm1reb} allele. A cassette containing negative (DTA) selection, positive selection (Neomycin, Neo) and lacZ was inserted into exon2 with a 5' splice acceptor (sa) sequence. B=BamHI, K=KpnI. (B) β-galactosidase activity in *Tarbp2* $^{tm1Reb/+}$ embryos from E8.5 to E13.5 stage of development. (C) β-galactosidase activity in sections of E13.5 organs of *Tarbp2* $^{tm1Reb/+}$ transcriptional reporter mice (blue= β-gal, red=nuclear fast red). Top and bottom insets show higher magnification images of indicated genotypes. Scale bar, 200um. RT-PCR in wild-type E13.5 organs total RNA extracts for *Tarbp2* (M/H Brain, mid/hind brain). (D) β-galactosidase activity in cross-sections of adult testis of *Tarbp2* $^{tm1Reb/+}$ transcriptional reporter mice (blue= β-gal, red=nuclear fast red). Top insets show higher magnification images of *Tarbp2* expression. Scale bar - 200um. (E) Alizarin red (bond) and alcian blue (cartilage) staining of E15.5 wild type and *Tarbp2* $^{-/-}$ B6 embryos showing defects in developmental patterning. (F) microCT scans of E15.5 wild-type and *Tarbp2* $^{-/-}$ B6 embryos, red outline shows smaller volume in mutant.

Supplemental_Fig_S1

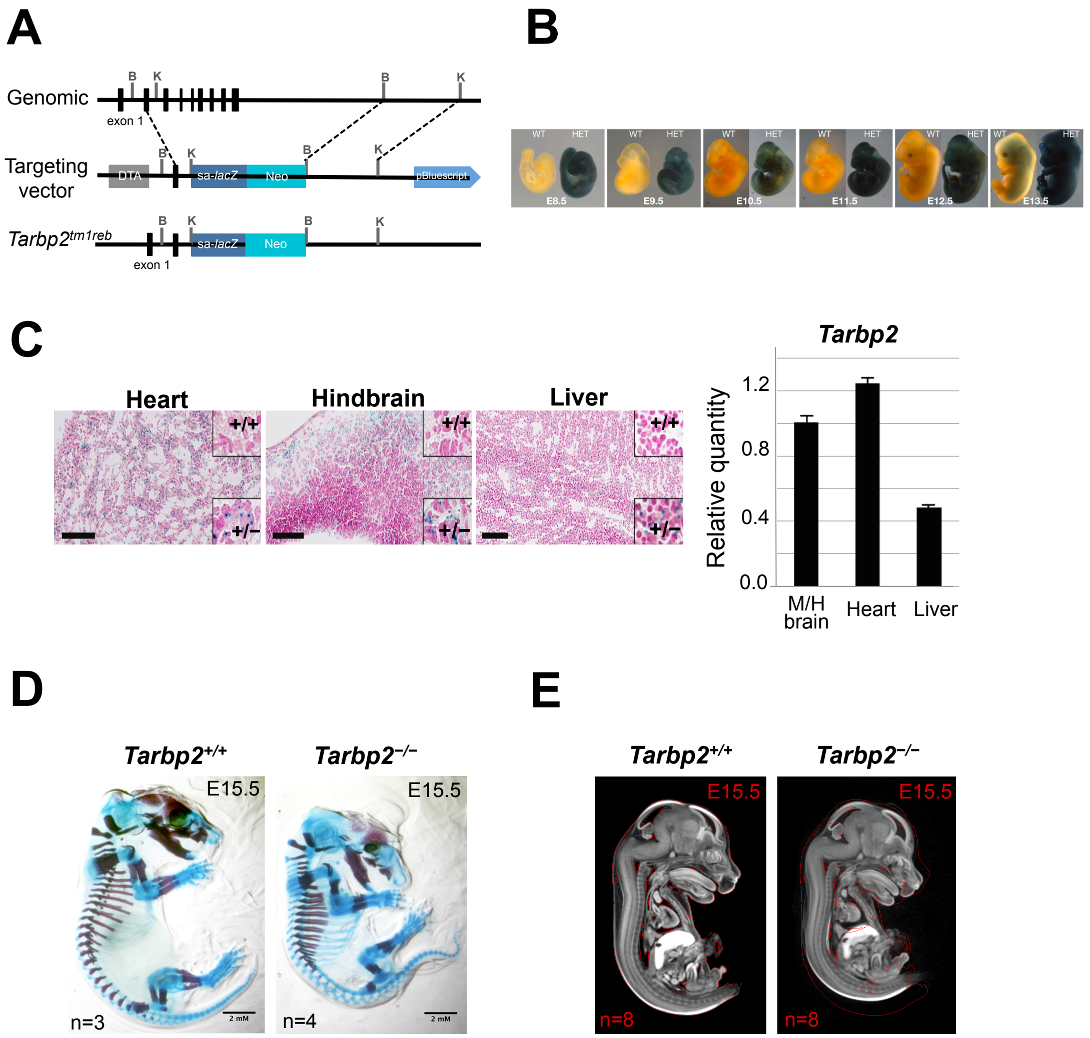

Supplement: Supplementary file 1 [file 1513FigureS1.pdf]
