# Supplementary material for: Functional Redundancy of DICER Cofactors TARBP2 and PRKRA During Murine Embryogenesis Does Not Involve miRNA Biogenesis
Source: Genetics. 2018 Feb 21;208(4):1513–22. doi: 10.1534/genetics.118.300791 (PMC5887145; doi:10.1534/genetics.118.300791)

**Supplemental_Fig_S2, related to Fig. 1.** (A) Summary of genomic/protein locations of the spontaneous *learJ* (little ears, The Jackson Laboratory) *Prkra* allele (*1j*), and an engineered insertion in exon (ex) 8 (*tm1Gsc*). (B) RT-PCR of *Prkra* transcripts in E13.5 organ extracts show significant decreases (*p-value<0.05) in heart and kidney. (C) Immunoblot for PRKRA shows decreased protein levels in all organs analyzed. (D) MicroCT images of skull showing immature fusion and opening of cranial sutures. RT-PCR in wild-type E13.5 organs total RNA extracts for *Prkra* (M/H Brain, mid/hind brain).

Supplemental_Fig_S2

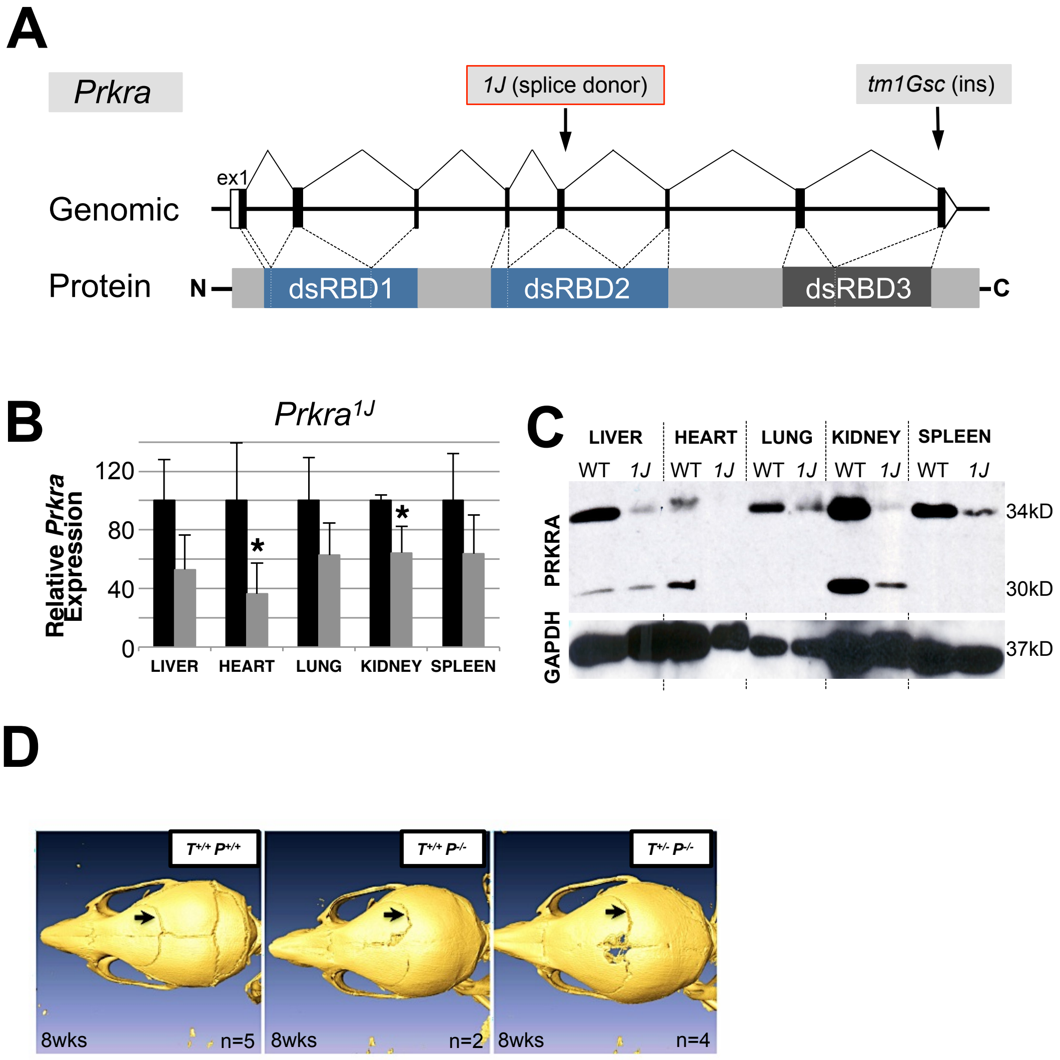

Supplement: Supplementary file 2 [file 1513FigureS2.pdf]
