# Supplementary material for: Functional Redundancy of DICER Cofactors TARBP2 and PRKRA During Murine Embryogenesis Does Not Involve miRNA Biogenesis
Source: Genetics. 2018 Feb 21;208(4):1513–22. doi: 10.1534/genetics.118.300791 (PMC5887145; doi:10.1534/genetics.118.300791)

**Supplemental_Fig-S3, related to Fig.4. :** Graphical representation of cell numbers calculated from Day 0 to Day 5 upon Enoxacin and DMSO treatment for each of the indicated genotype. The number of cells is the mean of four different experiments performed in triplicates±SD.

Supplemental_Fig_S3

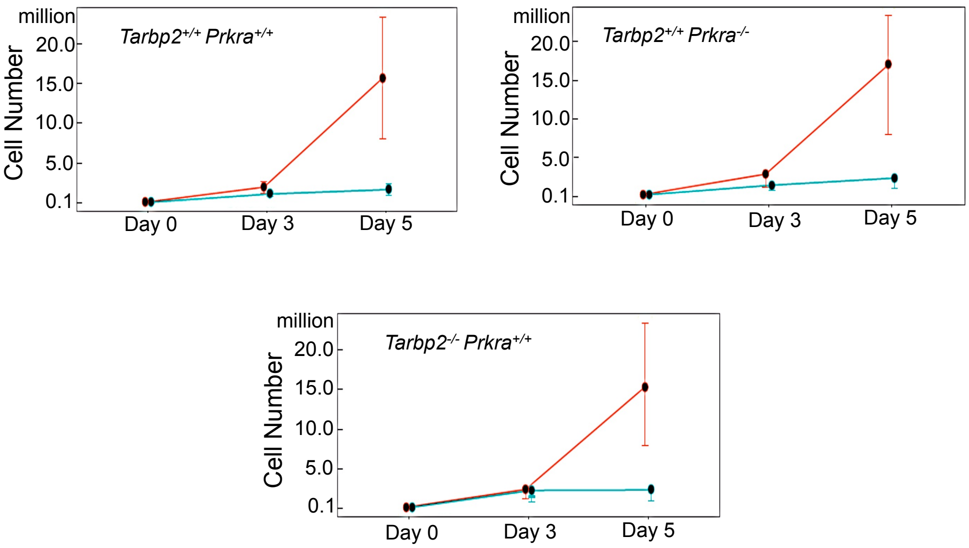

Supplement: Supplementary file 3 [file 1513FigureS3.pdf]
