# Supplementary material for: Functional Redundancy of DICER Cofactors TARBP2 and PRKRA During Murine Embryogenesis Does Not Involve miRNA Biogenesis
Source: Genetics. 2018 Feb 21;208(4):1513–22. doi: 10.1534/genetics.118.300791 (PMC5887145; doi:10.1534/genetics.118.300791)
Supplement: Supplementary file 4 [file 1513FigureS4.pdf]

Supplemental_Fig_S4: (A) Schematic representation of genetic crosses made to generate 129S1B6(F1) and 129S1B6(N2) to perform quantitative trait location analysis. (B) Kaplan-Meier curve showing the percentage survival of *Tarbp2*$^{-/-}$ animals with the progression of age on 129B6 mixed background compared to *Tarbp2*$^{+/-}$ and *Tarbp2*$^{+/+}$. (C) R/QTL maps using a binary variable that defined bodyweight and survival.

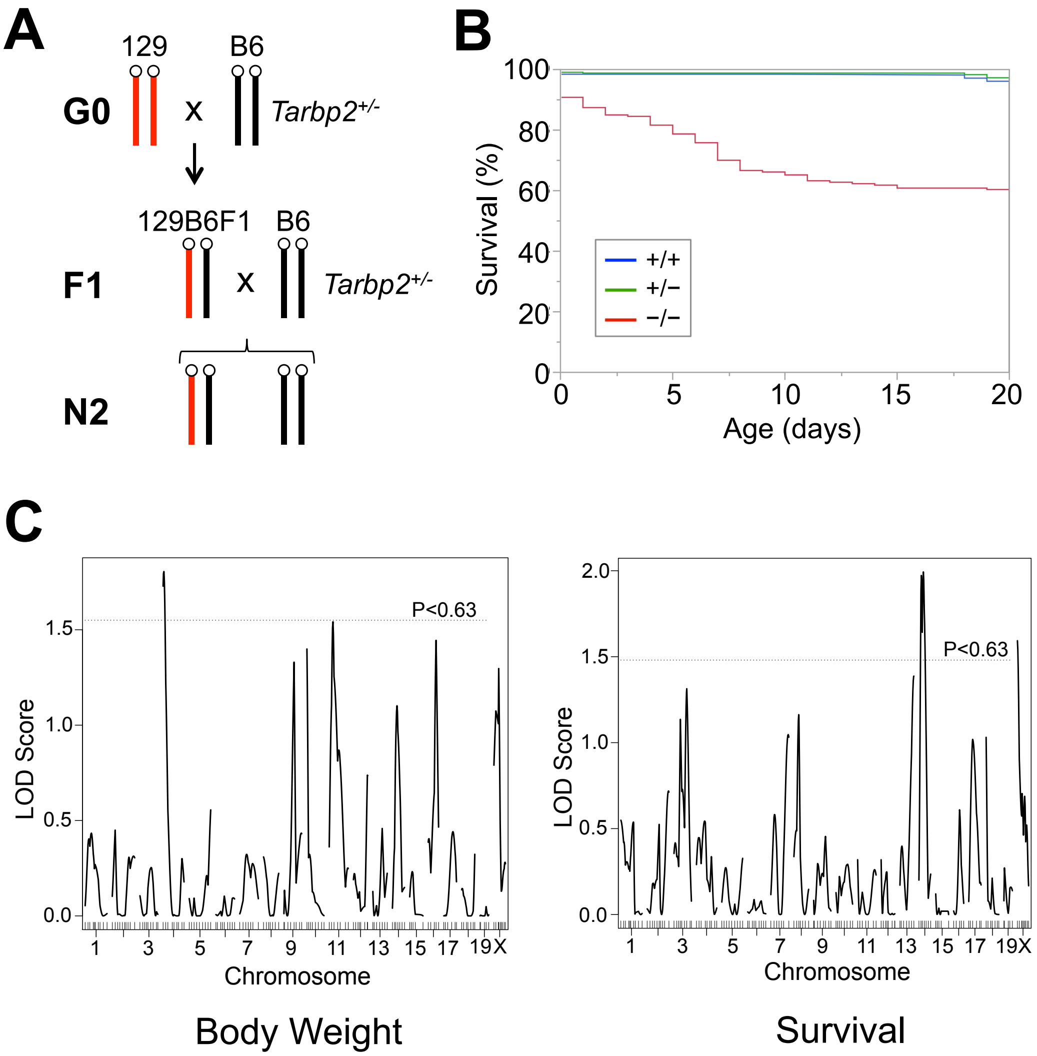
A
129
B6
G0
X
Tarbp2+/-
129B6F1
B6
F1
X
Tarbp2+/-
N2
B
100
80
60
40
20
0
Survival (%)
+/+
+/−
−/−
0
5
10
15
20
Age (days)
C
LOD Score
P<0.63
Chromosome
Body Weight
P<0.63
Chromosome
Survival
